# Creating a communication space in the healthcare context: Children’s perspective of using the eHealth service, Sisom

**DOI:** 10.1177/1367493520904804

**Published:** 2020-02-12

**Authors:** Ing-Marie Carlsson, Susann Arvidsson, Petra Svedberg, Jens M Nygren, Åsa Viklund, Anna-Lena Birkeland, Ingrid Larsson

**Affiliations:** 1 School of Health and Welfare, Department of Health and Nursing, Halmstad University, Halmstad, Sweden; 2 Department of Social Work, Astrid Lindgren Children’s Hospital, Karolinska University Hospital, Stockholm, Sweden

**Keywords:** Child-centred care, communication, eHealth, grounded theory, implementation

## Abstract

According to the United Nation’s Convention of the Rights of the Child, children have the right to participate in their own healthcare and make their opinions heard. The aim of this study was thus to explore the impact of using an eHealth service, Sisom, to gain the children’s perspectives during their healthcare appointments. Data were gathered through individual interviews with a purposeful sample of 16 children, aged 6–13 years old, treated for different diseases and using the eHealth service, Sisom, during their healthcare appointments. The interviews were analysed using a constructivist grounded theory. The results showed that using Sisom made children’s voice heard by creating a communication space in the healthcare setting. This meant that the children got involved in the communication, were acknowledged as an important person who could give the answers to questions and were given time. Implementing the use of Sisom is a way to make children’s needs and preferences explicitly visible for decision-making in practice and thereby supporting the further development of child-centred care in practice.

## Introduction

Children have the right to participate in their own healthcare and make their opinions heard (United Nations, 1989) which is also regulated by the Swedish Patient Act (SFS2014:821, 2014). However, the interpretation of what is conceptually meant by participation is sometimes vague and viewed upon as something that is implicit in the professional roles and the hospital’s organizations. Although healthcare professionals at paediatric clinics state that participation is a prerequisite for giving care (Carlsson et al., 2018), research concludes that children are only involved in minor and trivial matters regarding their care (Carlsson et al., 2018; Imms et al., 2016; van Bijleveld et al., 2015; Wyatt et al., 2015). Benefits from children’s involvement in healthcare have been shown, for example, feelings of being valued, increased sense of control and less anxiety (Coyne, 2006; Coyne and Harder, 2011; Dixon-Woods et al., 2002; Tornqvist et al., 2015). Healthcare organizations would thus benefit from adopting a child-centred approach that recognizes the child as an active participant in healthcare situations and that decisions should be based on the child’s experiences, perceptions and preferences (Coyne et al., 2016).

## Background

Communication and interaction with healthcare professionals are crucial for children’s participation in their own healthcare (Gilljam et al., 2016). Children seek involvement in consultations and generally prefer to have a say in issues that affect their health and healthcare (Coyne, 2006; Coyne and Harder, 2011). However, research demonstrates that children have a marginal role in discussions of their care (Cahill and Papageorgiou, 2007; Coyne, 2006; Moore and Kirk, 2010; Savage and Callery, 2007). Sometimes children are only involved in social conversations or even worse, completely left outside communication during consultations (Cahill and Papageorgiou, 2007).

Moreover, healthcare professionals often fail to provide opportunities for children to share their views regarding their care (Koller, 2017). There is thus a need for innovative solutions that support children’s participation in paediatric care as well as for strategies for implementation of such solutions (Armoiry et al., 2018; McNaughton and Light, 2013).

Thus, an implementation project with an eHealth service was performed in Sweden. Three different hospitals at four paediatric care centres were included in the implementation (Svedberg et al., 2019). The eHealth service, which was in the form of a digital interactive assessment and communication tool, was designed to facilitate children’s needs, that is, Sisom (Norwegian acronym ‘Si det som det er’ or ‘Tell it how it is’). Sisom has been developed together with children and has a child-friendly interface. In the form of a self-designed avatar, the child goes on a virtual journey travelling between 5 Islands with 84 animated questions. The questions are related to dimensions relevant to describing the children’s life situation and symptoms (Arvidsson et al., 2016; Ruland et al., 2008). The intention with Sisom is to capture the children’s perspective on their life situation and symptoms and have this as a basis for a dialogue with healthcare professionals (Baggott et al., 2015). No evaluation of children’s experiences of using Sisom in clinical practice has as yet been performed, and the current qualitative study is thus part of the evaluation of implementation of Sisom.

## Aim

To explore the impact of using an eHealth service to gain the children’s perspectives during their healthcare appointments.

## Method

### Design

A constructivist grounded theory was used since this method is well-suited to explore processes such as the implementation project evaluated in this study (Charmaz, 2014). The method acknowledges subjectivity, underlining that the participant’s experiences and realities are constructions influenced by the history and the cultural context in which they appear.

### Setting and intervention

The four included paediatric care centres differed somewhat in terms of hospital size, care delivery processes and the range of diagnoses treated at the centre. Two of the paediatric care centres were counsellor outpatient and inpatient care units, one was a paediatric oncology outpatient care unit and one was a paediatric neurology outpatient care unit.

The original version of Sisom has been tested for usability (Baggott et al., 2015; Ruland et al., 2008; Tsimicalis et al., 2014; Vatne et al., 2010, 2013). However, to better meet the needs of children of today, Sisom was recently redesigned for use on mobile devices, validated and adapted for use in a Swedish population of children (Arvidsson et al., 2016). The redesigned version of Sisom was used in this study.

The intervention with the eHealth service lasted for six months. During this time, each child used Sisom at least two times during their appointments. The children reported their self-assessments and feelings with the eHealth service by selecting the level of severity on a five-point Likert-type scale with cartoon faces (differently coloured smileys). The questions in Sisom were presented by spoken text, sound animations and intuitively meaningful metaphors and pictures. This variety made it possible for younger children who couldn’t read to understand and communicate through Sisom. After the child had completed Sisom, healthcare professionals printed the resulting summary and gave a copy to the child. This report then formed the basis for a forthcoming dialogue between professionals and children (Arvidsson et al., 2016; Ruland et al., 2008).

### Participants and data collection

A purposeful sampling was applied in this study, and 17 children were asked to participate. One child declined and 16 agreed to participate. The children were asked to participate by the nurses or social workers who had met the child during their appointments at the three different hospitals taking part in the implementation project.

The children who participated were 6–13 years old (eight girls and eight boys) and were treated for various forms of cancer, diabetes, heart diseases, hematologic diseases, HIV infections and neurological disease. Data were collected using unstructured interviews in order to capture the views of their experiences of using Sisom during their appointment at the hospital. The children were asked to talk freely about their experiences. Analysis and data collection were carried out simultaneously in accordance with the method (Glaser and Strauss, 1967), and questions pertaining to the emerging theory were asked during interviews. The interviews were undertaken by a researcher who didn’t have a pre-existing or ongoing relationship with the child or the parent. The interviews lasted between 30 minutes and 60 minutes and were undertaken either in the children’s homes, at the hospital, at the university or by videoconferencing tools (Skype or FaceTime). The interviews were audio-recorded and transcribed verbatim after each interview.

### Data analysis

The transcribed interviews were read carefully, and in the initial coding process, lines or segments of data with relevance for the aim were termed as condensed in vivo codes. The in vivo codes were written as active codes, keeping close to data by retaining the children’s words intact in the analysis process. By keeping close to the data, the children’s voice and meaning were maintained and present in the theoretical outcome (Charmaz, 2014). The next step in the analysis was the focused coding when the in vivo codes were compared, synthesized and pieced together based on similarities to larger segments of conceptualization. Together they formed a pattern of constructed concepts of categories and a core category, *making my voice heard*, emerged which formed the pivot around which all the categories were related. Finally, theoretical sampling was conducted, and additional data were sought to clarify some aspects and relations of the constructed set of categories and to confirm saturation of the identified categories. This was undertaken until all levels of categories appeared complete, that is, no new data were found to indicate new categories or expansion of existing categories. During the whole analysis process, a constant comparison of each level of analysis was used to refine the data, relations and interrelations and memos were written, and a final step was the sorting of memos that were used and integrated into the theory.

### Ethical considerations

The study was approved by the Regional Ethical Review Board at Lund University, Sweden (approval numbers REF 2015/174 and 2016/189), and the study conforms to the principles outlined in the Declaration of Helsinki (WMA, 2013). The participating children and their parents received written and verbal information about the study. They were informed that all data would be treated confidentially, and to protect participant identity, pseudonyms would be used. Further, they were ensured that their participation or non-participation had no impact on their care and were guaranteed that they could withdraw their participation whenever they wanted. The parents signed consent forms and chose the timing and place for the interviews themselves.

## Findings

### The theory of making children’s voices heard, by creating a communication space

The basic social process, making children’s voices heard by creating a communication space in the healthcare setting, explained the main impact of Sisom from the children’s perspectives when it was used during hospital appointments. The children generally perceived the hospital appointments as a stressful event although there was, however, an acceptance that they had no choice and had to endure this to be cured. According to the children’s narratives, their involvement in the communication or in the decisions made about their care was marginal. They described only receiving a few questions that were directed specifically towards them and did not choose or influence what they were talking about to any great extent. However, this changed when they used Sisom.

A communication space was created using Sisom, which entailed that the child was involved in the communication, was acknowledged as an important person who could give answers to questions and was given time. The communication space thus improved the opportunity for an exchange of thoughts and ideas between the children, the parents and the healthcare professionals, which enabled a greater understanding and a higher level of participation for the children.

**Figure 1.**
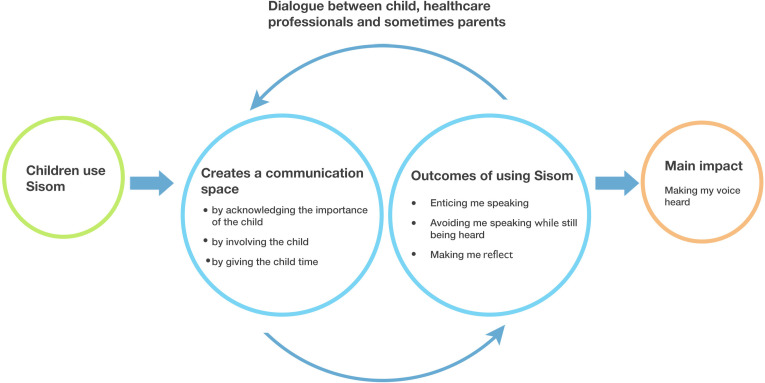
The basic social process, making children’s voice heard by creating a communication space in the healthcare setting.

The created communication space together with three subcategories: (1) *enticing me to speak*, (2) *avoiding speaking while still being heard* and (3) *making me reflect*, that were outcomes of the use of Sisom, accounted for a pattern of relevance of the implemented interactive assessment and communication tool. This pattern enabled and strengthened the core category, *making my voice heard*, that was found to be the main concern and main impact of using Sisom, for the children involved (see Figure 1).

### The three subcategories

The three subcategories *enticing me to speak, avoiding speaking while still being heard* and *making me reflect* enabled the children to voice their opinions on aspects of care, which made parents and healthcare professionals listen to them.

#### Enticing me to speak

The children expressed that using Sisom enticed them to speak instead of letting their parents speak. There was an excitement when using technical and mobile devices which the children were familiar with. The use of mobile devices and animated applications was expressed as always being fun and interesting, and Sisom was described as enjoyable. When using Sisom, the children had to start by creating an animated, personalized avatar, the main character. This character was the one who travelled around the islands on a boat and answered questions that were asked on the different islands. The children spoke of identifying themselves with the avatar and that they imagined them being the avatar, which was far more entertaining than ordinary verbal talk that was seen as boring.

I think it’s fun. It might not be. It’s fun to click on it. It’s always fun with a tablet. Then you’ve got something to do. Something happens. And you can create your own person. I think that’s fun. (Alice)

Furthermore, Sisom was described as uncomplicated and easy to use. Applying a mobile device and just be able to do a ‘click’ was perceived as doing something in a simpler way, which made communication more relaxed, informal and easier. It was also sometimes difficult to talk about themselves or about certain things, and when Sisom was used, the children sometimes received fewer difficult follow-up questions.

It feels good because it can’t be that she asks what do you mean or what are you thinking when you answer like that and have to talk about that. // Because you may not want to explain how you meant, you just want to say what say it’s like this. And not get any more questions that they want to know why or in which way you’re thinking. (Theodor)

The mobile device with the application Sisom was perceived by the children as making it easier to concentrate and express themselves. Even if the questions presented on the screen were frightful or inconvenient the children preferred this non-verbal and fun way of communicating compared to answering questions verbally or on paper. Sisom contained five animated islands, where the focus in the questions varied. Some of them had questions related to school or their diseases and various matters that might be frightening, such as questions about teasing or bullying at school and tough questions about dying. Nonetheless, the children considered these questions as something that were important to talk about and they were willing to respond because the healthcare professional could perhaps help and improve things for the child. Even though these questions were generally more difficult to talk about and could be upsetting or problematic to give a reply to, they became easier to answer when using Sisom due to the communication tool enticing them and giving them the courage to speak up.

Furthermore, the children acknowledged that Sisom also asked new questions that nobody had asked before and which they probably had not talked about unless Sisom had asked the questions. They stated that they never felt obligated to answer the questions, instead, the mobile device gave them the opportunity to stop responding without feeling pressured.

It perhaps became a little better because you don’t questions all the time, yes it’s much more fun sitting with tablet so I think it’s good they did that. Then you get to click on a tablet. (Alice)

because it’s fun to click and one can answer, it’s easier than talking. (William)

The children thought Sisom made it easier to explain their situation to the healthcare professionals. Some of them also told the family what they responded to in Sisom and they said that it was fun to tell the family.

I thought it was good because it was easier to say what I wanted on an app. And that they talk about it afterwards. (Bianca)

Furthermore, the answers that were given by the child could be printed in a report and used in the communication during the appointments or at home with the parents. The children acknowledged that they noticed that the healthcare professionals used the child’s answers in Sisom and talked more about specific areas where they had used a red smiley that indicated that they had problems with the area in question. Some of the children used their report of the answers in Sisom to speak with their parents at home.

I thought it was good so that I could explain why I chose that (the answers to the questions in Sisom (Oliver)

#### Avoiding speaking while still being heard

The children could avoid inconvenient situations when using Sisom and remain silent while still being heard. The children described that the hospital appointments as being stressful for them and that it was difficult to go to the hospital and that they did not want to be there. Being in a hospital and facing healthcare professionals, who were sometimes unknown people, could cause anxiety and an unwillingness to talk.

I’m able to say what I want but avoid talking, that’s how I feel about it….Because sometimes it can be, it can be hard to say it…sometimes you don’t want to say exactly what you’re thinking. (Ellie)

Facing questions from the healthcare professionals could be considered difficult to avoid and to decline to speak about. The situation could be uncomfortable, especially when the child received follow-up questions that they did not want to answer.

For some people are perhaps afraid for things and don’t want to say things directly. Then they can click on the thing they’re afraid of. It’s easier to click than to talk. It feels good to avoid talking. (Stella)

Furthermore, avoiding sometimes meant that the children indicated that they were not in the mood for talking. The category illustrates how Sisom reduced the discomfort that the child could associate with the face to face contact. Instead, using Sisom the child could just keep the information for themselves, avoiding to say directly what they thought and remaining silent while still being heard. This was interpreted as empowering and created a sense of daring.

I just think it was a good app for me as a child who for example, who finds it a little harder to talk about problems but not perhaps not so hard to talk about other things. So it’s good that there is such an app that helps children. (Bianca)

The children could decide if they wanted to share their answers with the family or not. This was considered helpful since the information could be shared without talking. One of the children chose to keep the answers secret from the family but chose to answer questions in Sisom several times. Some children said that their answers to the questions in Sisom could have been different if the healthcare professionals or parents had not been around.

No, it was harder to say, that is answer truthfully or so because you knew that she (the member of staff) sat there and checked. // You wonder what she or he thinks about this. (Theodor)

…it’s difficult to speak, to speak out, I think that’s hard sometimes. But most often it’s OK. It can be hard to say things, things that you don’t want to say or you just don’t want to say it because it’s a bit, how can I explain…? (Alice)

The healthcare professionals respected the desire of a child who wanted the answers to be secret. Some of the children did not always want to or had the strength to talk about a particular question in Sisom, sometimes they made a conscious choice to not answer honestly to all the questions that resulted in the child continuing to feel bad

I answered mostly and clicked // to get it over with // it was more because I couldn’t talk anymore about it. // Then I filled in this many smiley faces. (Ellie)

If you don’t answer truthfully to all the questions and say that everything is OK even though it isn’t. Then you carry on feeling bad and that’s not good in the long run. (Ellie)

#### Making me reflect

Time and space were created for the children when Sisom was used during their appointment at the hospital. The children were allowed to sit down and answer the questions without stress, which helped to calm them. They could either chose to do it by themselves or if they preferred it together with a healthcare professional or a parent beside them. Sisom enabled them to use different senses, both hearing and reading the questions and viewing the visual virtual animations. This gave them time for consideration and reflection before given the answers. Enabling this resulted in more, well-deliberated answers, which they considered was different from when they were usually asked verbal questions during their appointment and had to answer quickly to the healthcare professional.

…you could do it in peace and quiet (complete Sisom) and it wasn’t just to answer quickly but you could think it through and didn’t need to be super-involved. You didn’t need to be pressed to answer (Noah)

it’s easier to look at the questions and then think about them instead of hearing them. Then you can think yourself in your own head. (Adrian)

Moreover, using Sisom, they were asked questions that nobody had asked before, which meant that they had to have a break and think about the question a little longer before they gave an answer. The children were very meticulous about how they answered the questions, as they did not want the answer to be interpreted incorrectly by the healthcare professional and their parents. The children were made to reflect on their health and memories of the process they had gone through when Sisom was used more than once. A child expressed that she had a favourite island that was called ‘managing things’. By comparing her answers about how she had been able to hold a pen before, made her realize that she had improved her ability quite a lot. This reflection created a sense of warmth and of being more self-efficient.

I discussed things that came up in Sisom that I hadn’t talked so much about. So I became better at doing that anyway. (Ellie)

The questions in Sisom generated thoughts and reflections in the children, and it is important that healthcare professionals pay attention to this. However, the children thought it was important to see Sisom as a communication tool and that a more profound follow-up communication should be done by healthcare professionals.

I had more of these personal matters that I wanted to talk about but that weren’t in Sisom. Yes, so I brought it up, because I wanted to talk about it. Then we had to prioritize that instead of Sisom. (Ellie)

## Discussion

The result from this study show the children’s experiences of using Sisom made their voices heard, by creating a communication space with both the healthcare professionals and the parents. The use of Sisom acknowledged the importance of the children, involved the children and gave them time to express their feelings and thoughts and, thus, the communication space was created. Sisom enticed the children to speak, allowed them to avoid speaking while still being heard and made them reflect and provide answers that were more deliberated. Moreover, the children’s willingness to answer questions was improved by Sisom, while at the same time reducing the feeling of being pressured. They were more in charge of the information and could choose if the parents could take part of their experiences and thoughts or not. Even if difficult questions became easier to answer in the fun way provided by Sisom, they were able to express negative feelings such as disappointment. The result from this study thus indicates that Sisom can be a useful tool for meeting the requirement that all children have the right to be listened to and participate in decision-making about their own healthcare (United Nations, 1989).

There is an explicit need for the development of appropriate, child-friendly eHealth solutions in paediatric settings in order to address children’s right to participation (Desai and Pandya, 2013). However, evidence on the outcomes of using such eHealth solutions developed with and for children in healthcare is sparse. While global research on the development of e-health solutions has increased exponentially, few studies have explored children’s perspective on their needs, preferences and experiences of such solutions (Larsson et al., 2018). Children’s participation has only been addressed in a few studies (Raaff et al., 2014), and no such eHealth solutions have been implemented in practice (Enam et al., 2018). Based on the importance of eliciting children’s experiences, needs and preferences for providing a child-centred approach in the context of paediatric healthcare (Coyne et al., 2016; Soderback et al., 2011), we found that Sisom from the child’s perspective was a relevant and appropriate digital tool. One explanation for this positive result can be that Sisom has been developed together with children, based on their needs on content, questions, aesthetics and the visual interface as well as usability (Arvidsson et al., 2016) and not only based on the healthcare professionals’ perspectives on children’s needs. This is interesting as most research and innovation projects with the purpose of developing interventions for children are primarily based on the involvement of parents, healthcare professionals and other stakeholders (Nygren et al., 2017). We thus need to recognize the value of seeking children’s views and their capacity to participate in the development of e-health solutions. Parents and healthcare professionals’ perspectives cannot replace the qualities that come with genuine participation by the children themselves.

The use of Sisom gave the children time to express their feelings and thoughts, changed the communication pattern between the children and the healthcare professionals and strengthened the children’s empowerment. The findings of the study have confirmed that children have much to offer healthcare professionals by expressing their views about a range of health-related matters that concern them. Children’s views can support healthcare professionals in understanding the child’s perspective on their situation and thus increase the possibility for shared decision-making of appropriate interventions and treatments. Facilitating children’s involvement in decisions hopefully creates a sense of control for them and may increase the possibility that the child will gain greater empowerment in the future (Grootens-Wiegers et al., 2017; Miller, 2018). Children want to be spoken to and be active in their conversation with healthcare professionals (Gibson et al., 2010), but they need to have support, appropriate communication tools and time to practice before being able to make effective decisions on their own. However, it is important to notice that the children in our study spoke of sometimes not having enough strength to express their opinions and that Sisom helped them to be heard without having to speak aloud. Similarly, children describe that they sometimes want to leave decisions to their parents or healthcare professionals (Coyne and Gallagher, 2011; Coyne et al., 2014; Gibson et al., 2010; Wangmo et al., 2017). It is thus important to create a communication space where it is possible for the children to have a dialogue about how they want to be included in the conversation and if and how they want to participate in decision-making in the specific situation. A movement towards more child-appropriate, digital communication tools, such as Sisom, could probably affect the expectations and the role of the child, the parents and the healthcare professionals and thus facilitate a more child-centred care.

## Methodological considerations

Grounded theory implies that the researcher should do research with an opened clear mind, with as few previous predetermined ideas as possible (Glaser, 1978). In this study, the authors who performed the analysis didn’t take part in the development or the implementation process of the eHealth service, Sisom. This could be argued as a very first step in gaining theoretical sensitivity. In addition, later on, all authors discussed the emerging concepts and their relationships, opening up the analysis for scrutiny, which strengthened the theoretical sensitivity further. Another strength is that the eHealth service was implemented and studied at four paediatric care centres that differed in terms of hospital size, care delivery processes and the range of diagnoses treated at the centre. However, the sample was children who had several visits at the centres, and further research is needed into when it is optimal to use the eHealth service and the eligibility for global use.

## Implications for practice

According to Convention on the Rights of the Child (United Nations, 1989), children have the right to be listened to in all situations that affect them. This could be facilitated in clinical practice by routinely using an eHealth solution such as Sisom in paediatric healthcare.

## Conclusion

The use of eHealth solutions for improved communication is a developing field in paediatric healthcare, which has strong implications for children, parents and healthcare professionals. The findings showed that Sisom created a communication space in the healthcare context. This space entailed that the child was involved in the communication, was acknowledged as an important person who could give answers to questions and was given time. The communication space thus enabled the children to voice their opinions on aspects of care which made the parents and the healthcare professionals listen to them and enabled a greater understanding and a higher level of participation for the children. Our results showed that Sisom can strengthen children’s empowerment and support the requirements for developing ways to make children’s needs and preferences explicitly visible in decision-making in practice and thus support the ambition of furthering the development of child-centred care in practice.

## References

[bibr1-1367493520904804] Armoiry X Sturt J Phelps EE, et al. (2018) Digital clinical communication for families and caregivers of children or young people with short- or long-term conditions: rapid review. Journal of Medical Internet Research 20: e5. 29305339 10.2196/jmir.7999 PMC5775486

[bibr2-1367493520904804] Arvidsson S Gilljam BM Nygren J, et al. (2016) Redesign and validation of Sisom, an interactive assessment and communication tool for children with cancer. JMIR mHealth uHealth 4: e76. 27343004 10.2196/mhealth.5715 PMC4938887

[bibr3-1367493520904804] Baggott C Baird J Hinds P, et al. (2015) Evaluation of Sisom: a computer-based animated tool to elicit symptoms and psychosocial concerns from children with cancer. European Journal of Oncology Nursing 19: 359–369. 25691300 10.1016/j.ejon.2015.01.006

[bibr4-1367493520904804] Cahill P Papageorgiou A (2007) Triadic communication in the primary care paediatric consultation: a review of the literature. British Journal of General Practice 57: 904–911. 10.3399/096016407782317892 PMC2169315 17976292

[bibr5-1367493520904804] Carlsson IM Nygren JM Svedberg P (2018) Patient participation, a prerequest for care: a grounded theory study of healthcare professionals’ perceptions of what participation means in a paediatric care context. Nursing Open 5(1): 45–52. 29344394 10.1002/nop2.106 PMC5762705

[bibr6-1367493520904804] Charmaz K (2014) Constructing Grounded Theory. Thousand Oaks, CA: Sage.

[bibr7-1367493520904804] Coyne I Gallagher P (2011) Participation in communication and decision-making: children and young people’s experiences in a hospital setting. Journal of Clinical Nursing 20: 2334–2343. 21410571 10.1111/j.1365-2702.2010.03582.x

[bibr8-1367493520904804] Coyne I Harder M (2011) Children’s participation in decision-making: balancing protection with shared decision-making using a situational perspective. Journal of Child Health Care 15: 312–319. 21828162 10.1177/1367493511406570

[bibr9-1367493520904804] Coyne I Amory A Kiernan G, et al. (2014) Children’s participation in shared decision-making: children, adolescents, parents and healthcare professionals’ perspectives and experiences. European Journal of Oncology Nursing 18: 273–280. 24629505 10.1016/j.ejon.2014.01.006

[bibr10-1367493520904804] Coyne I Hallstrom I Soderback M (2016) Reframing the focus from a family-centred to a child-centred care approach for children’s healthcare. Journal of Child Health Care 20(4): 494–502. 27141084 10.1177/1367493516642744

[bibr11-1367493520904804] Coyne I (2006) Consultation with children in hospital: children, parents’ and nurses’ perspectives. Journal of Clinical Nursing 15: 61–71. 16390525 10.1111/j.1365-2702.2005.01247.x

[bibr12-1367493520904804] Desai PP Pandya SV (2013) Communicating with children in healthcare settings. The Indian Journal of Pediatrics 80: 1028–1033. 23378054 10.1007/s12098-013-0969-z

[bibr13-1367493520904804] Dixon-Woods M Anwar Z Young B, et al. (2002) Lay evaluation of services for childhood asthma. Health & Social Care in the Community 10: 503–511. 12485138 10.1046/j.1365-2524.2002.00390.x

[bibr14-1367493520904804] Enam A Torres-Bonilla J Eriksson H (2018) Evidence-based evaluation of eHealth interventions: systematic literature review. Journal of Medical Internet Research 20: e10971. 30470678 10.2196/10971 PMC6286426

[bibr15-1367493520904804] Gibson F Aldiss S Horstman M, et al. (2010) Children and young people’s experiences of cancer care: a qualitative research study using participatory methods. International Journal of Nursing Studies 47: 1397–1407. 20430388 10.1016/j.ijnurstu.2010.03.019

[bibr16-1367493520904804] Gilljam BM Arvidsson S Nygren JM, et al. (2016) Promoting participation in healthcare situations for children with JIA: a grounded theory study. International Journal of Qualitative Studies on Health and Well-being 11: 30518. 27172512 10.3402/qhw.v11.30518 PMC4864848

[bibr17-1367493520904804] Glaser BG Strauss A (1967) The Discovery of Grounded Theory: Strategies for Qualitative Research. New York: Aldine de Gruyter.

[bibr18-1367493520904804] Glaser BG (1978) Theoretical Sensitivity: Advances in the Methodology of Grounded Theory. San Francisco: The Sociology Press.

[bibr19-1367493520904804] Grootens-Wiegers P Visser EG van Rossum AMC, et al. (2017) Perspectives of adolescents on decision making about participation in a biobank study: a pilot study. BMJ Paediatrics Open 1: e000111. 29637137 10.1136/bmjpo-2017-000111 PMC5862224

[bibr20-1367493520904804] Imms C Adair B Keen D, et al. (2016) ‘Participation’: a systematic review of language, definitions, and constructs used in intervention research with children with disabilities. Developmental Medicine & Child Neurology 58: 29–38. 26411643 10.1111/dmcn.12932

[bibr21-1367493520904804] Koller D (2017) ‘Kids need to talk too’: inclusive practices for children’s healthcare education and participation. Journal of Clinical Nursing 26: 2657–2668. 28001330 10.1111/jocn.13703

[bibr22-1367493520904804] Larsson I Staland-Nyman C Svedberg P, et al. (2018) Children and young people’s participation in developing interventions in health and well-being: a scoping review. BMC Health Services Research 18: 507. 29954392 10.1186/s12913-018-3219-2 PMC6027768

[bibr23-1367493520904804] McNaughton D Light J (2013) The iPad and mobile technology revolution: benefits and challenges for individuals who require augmentative and alternative communication. Augmentative and Alternative Communication 29: 107–116. 23705813 10.3109/07434618.2013.784930

[bibr24-1367493520904804] Miller VA (2018) Optimizing children’s involvement in decision making requires moving beyond the concept of ability. American Journal of Bioethics 18: 20–22. 10.1080/15265161.2017.1418923 PMC6214154 29466131

[bibr25-1367493520904804] Moore L Kirk S (2010) A literature review of children’s and young people’s participation in decisions relating to health care. Journal of Clinical Nursing 19: 2215–2225. 20659201 10.1111/j.1365-2702.2009.03161.x

[bibr26-1367493520904804] Nygren JM Lindberg S Warnestal P, et al. (2017) Involving children with cancer in health promotive research: a case study describing why, what, and how. JMIR Research Protocols 6: e19. 28174150 10.2196/resprot.7094 PMC5320392

[bibr27-1367493520904804] Raaff C Glazebrook C Wharrad H (2014) A systematic review of interactive multimedia interventions to promote children’s communication with health professionals: implications for communicating with overweight children. BMC Medical Informatics and Decision Making 14: 8. 24447844 10.1186/1472-6947-14-8 PMC3926331

[bibr28-1367493520904804] Ruland CM Starren J Vatne TM (2008) Participatory design with children in the development of a support system for patient-centered care in pediatric oncology. Journal of Biomedical Informatics 41: 624–635. 18082468 10.1016/j.jbi.2007.10.004

[bibr29-1367493520904804] Savage E Callery P (2007) Clinic consultations with children and parents on the dietary management of cystic fibrosis. Social Science & Medicine 64: 363–374. 17064831 10.1016/j.socscimed.2006.09.003

[bibr30-1367493520904804] SFS2014:821 (2014) The Patient Act. Stockholm: The Swedish Government.

[bibr31-1367493520904804] Soderback M Coyne I Harder M (2011) The importance of including both a child perspective and the child’s perspective within health care settings to provide truly child-centred care. Journal of Child Health Care 15: 99–106. 21685225 10.1177/1367493510397624

[bibr32-1367493520904804] Svedberg P Arvidsson S Larsson I, et al. (2019). Barriers and enablers affecting successful implementation of the Electronic Health Service Sisom: multicenter study of child participation in pediatric care. Journal of Medicinal Internet Research (JMIR) 21(11): e14271. 10.2196/14271 PMC6884717 31730040

[bibr33-1367493520904804] Tornqvist E Mansson A Hallstrom I (2015) Children having magnetic resonance imaging: a preparatory storybook and audio/visual media are preferable to anesthesia or deep sedation. Journal of Child Health Care 19: 359–369. 24486815 10.1177/1367493513518374

[bibr34-1367493520904804] Tsimicalis A Stone PW Bakken S, et al. (2014) Usability testing of a computerized communication tool in a diverse urban pediatric population. Cancer Nursing 37: E25–E34. 24457227 10.1097/NCC.0000000000000115 PMC4547853

[bibr35-1367493520904804] United Nations (1989) Conventions of the Rights of the Child. UNICEF. Geneva: UN.

[bibr36-1367493520904804] van Bijleveld GG Dedding CWM Bunders-Aelen JFG (2015) Children’s and young people’s participation within child welfare and child protection services: a state-of-the-art review. Child & Family Social Work 20: 129–138.

[bibr37-1367493520904804] Vatne TM Finset A Ørnes K, et al. (2013) Effects of an interactive symptom communication tool for children with heart disease on patient-provider communication in outpatient care: preliminary results. Journal of Communication in Healthcare 6: 106–114.

[bibr38-1367493520904804] Vatne TM Slaugther L Ruland CM (2010) How children with cancer communicate and think about symptoms. Journal of Pediatric Oncology Nursing 27: 24–32. 19833978 10.1177/1043454209349358

[bibr39-1367493520904804] Wangmo T De Clercq E Ruhe KM, et al. (2017) Better to know than to imagine: including children in their health care. AJOB Empirical Bioethics 8: 11–20. 28949869 10.1080/23294515.2016.1207724

[bibr40-1367493520904804] WMA (2013) World Medical Association declaration of Helsinki – Ethical principles for medical research involving human subjects. JAMA 310: 2191–2194. 24141714 10.1001/jama.2013.281053

[bibr41-1367493520904804] Wyatt KD List B Brinkman WB, et al. (2015) Shared decision making in pediatrics: a systematic review and meta-analysis. Academic Pediatrics 15: 573–583. 25983006 10.1016/j.acap.2015.03.011

